# Some Exposure for Influenza Hemagglutinin Interface Antibodies

**DOI:** 10.1111/imr.70167

**Published:** 2026-08-26

**Authors:** Kevin R. McCarthy

**Affiliations:** ^1^ Center for Vaccine Research, University of Pittsburgh School of Medicine Pittsburgh Pennsylvania USA; ^2^ Department of Microbiology and Molecular Genetics University of Pittsburgh School of Medicine Pittsburgh Pennsylvania USA

## Abstract

Influenza viruses are a constant threat to human health. Current vaccines often confer low to moderate protection against infection with seasonal viruses and little‐to‐no protection against pandemic viruses. Improved vaccines are needed. These vaccines will need to reliably elicit antibodies to conserved sites that are broadly protective. One strategy to do so is to design immunogens that present epitopes that are targeted by antibodies that are genetically similar, that humans have the genetic capacity to produce, and that are likely present in human naive B cell repertoires. Among the more recently described epitopes for broadly binding, protective antibodies are those on surfaces at protein–protein interfaces. This review will focus on describing many of these sites and the genetic features of antibodies that engage them. Interface epitopes are immunogenic, and antibodies to these sites are likely present in human repertoires following influenza infection or vaccination.

## 1 Introduction

Historically, antigenically novel influenza viruses have emerged from animal reservoirs, caused pandemics, and continued to circulate as seasonal variants by acquiring resistance to immunity elicited by previous exposures [1]. Pandemic events have killed millions to tens of millions of people, while seasonal viruses kill hundreds of thousands annually [2]. Current influenza vaccines have moderate effectiveness against seasonal viruses and confer little‐to‐no protection against pandemic strains [3]. Improved vaccines that confer more broadly protective, lasting immunity are needed.

Antibodies confer the strongest protection against influenza virus infection [4, 5]. The established correlate of protection is the titer of serum antibodies required to block the influenza hemagglutinin (HA) protein from engaging its cellular receptor, cell surface sialic acids present on glycoproteins and glycolipids. These antibodies prevent HA from attaching to cells, preventing cell entry. Antibodies to a second glycoprotein present on the influenza virion surface, neuraminidase (NA), have also been shown to be protective in small animal models. NA facilitates virus egress by cleaving the glycosidic bond between terminal sialic acids and the carbohydrate chain to which they are appended.

**FIGURE 1:**
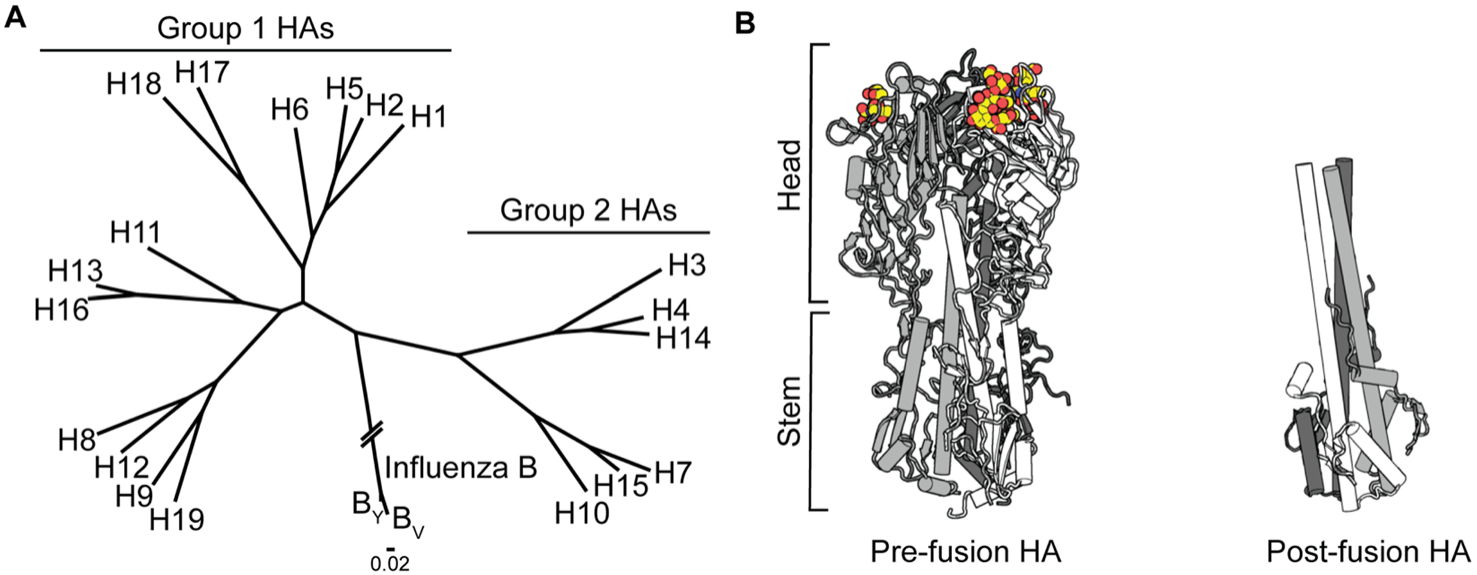
Influenza hemagglutinin is a major antigenic determinant. (A) A phylogenetic tree of influenza virus type A and B hemagglutinin proteins. They are labeled by their numeric subtypes and divided into group 1 and group 2 viruses, which occupy opposing sides of the tree. Influenza B viruses are classified into two lineages, B Yamagata (B_Y_) and B Victoria (B_V_). (B) Left: A structure of the pre‐fusion HA trimer (PDB 2VIU [6]). It is organized into a globular head and helical stem regions. Protomers are colored from white to dark gray. A tetrasaccharide terminating with a sialic acid is modeled (PDB 3UBE [7]) (carbon is yellow, oxygen red, and nitrogen blue). Right: A post‐fusion structure of HA (PDB 1HTM [8]). Protomers are colored from white to dark gray.

Antigenicity is intimately tied to influenza virus classification. The HAs and NAs of influenza are classified for their antigenic/genetic relatedness to known isolates. These sequentially numbered HA and NA types are used to broadly categorize isolates (e.g., H1N1, H3N2, or H5N8). Currently, there are 19 recognized subtypes of HA (Figure 1A) and 11 of NA. HAs and NAs freely reassort in nature to produce novel HxNy variants of unknown consequence.

Antibodies drive the evolution of seasonal influenza viruses in humans [9]. Influenza virus evolution is most rapid in HA, followed by NA [10]. Within HA, sites surrounding an otherwise conserved receptor binding site (RBS) have the most accelerated rate of evolution [9, 11, 12, 13]. Importantly, the “footprint” of an antibody to the RBS exceeds the contact surface of a carbohydrate chain that is terminated by a sialic acid [14].

Antibodies to conserved sites that do not have receptor blocking activity have been identified in human antibody repertoires, are broadly reactive, and are protective in small animal models [14]. The existence of these antibodies suggests that immunogens can be designed and immunization regimens optimized to reliably elicit polyclonal, broadly protective antibody responses in humans. Many of these antibodies engage solvent‐exposed surfaces on the lateral sides of the HA head (lateral patch), the vestigial esterase, central stem, and anchor [14, 15].

More recently, broadly protective antibodies to surfaces that are seemingly occluded in the understood prefusion form of HA have been identified [16, 17, 18, 19, 20, 21, 22, 23, 24]. These epitopes are at protein–protein interfaces buried within the HA trimer. Exposure of interface epitopes requires conformational rearrangements that may include transient molecular “breathing” events or a full transition of HA to its stable post‐fusion form. Due to evolutionary constraints, HA protein–protein interfaces are widely conserved, both within a single HA protomer, across interfaces in the HA trimer, and those that are exposed in the post‐fusion form of HA. Collectively, these antibodies are non‐neutralizing and have little‐to‐no impact on viral spread in vitro. However, when passively transferred in murine models of infection, these antibodies limit disease severity and enhance survival. These metrics are enhanced when they are delivered with an Fc region that can efficiently direct cell killing through cellular (phagocytic and natural killer) and complement‐mediated pathways. While these antibodies likely do not protect against infection, they may have the capacity to limit disease severity in humans, as they do in small animal models. Selectively eliciting interface antibodies may augment responses to other sites that directly protect against infection. This review will outline the genetic features of the known, interface‐directed antibody response to HA.

## 2 Hemagglutinin Undergoes Conformational Rearrangements to Infect Cells

HA forms a homotrimer. It is described to have a globular “head” region and a helical “stem” region (Figure 1B). The head harbors the receptor‐binding site, and the stem facilitates virus‐cell membrane fusion [25]. Influenza virions attach to cells by engaging glycans with specific, terminal, sialic acid linkages [1, 14, 25]. Upon encountering the appropriate endosomal pH, HA undergoes an orchestrated series of conformational rearrangements that result in the fusion of virion and cellular membranes and the deposition of the influenza virus genome in the target cell [25, 26]. The prefusion form of HA differs markedly from its post‐fusion form (Figure 1B) [8, 27]. Most of the stem region is rearranged, forming a stable six‐helix bundle with protomers in the trimer. While not currently resolved by high‐resolution structures, the globular HA heads transition from sitting upon the helical stem region to being displaced on a flexible linker that is tethered to the six‐helix bundle via a disulfide bond [26].

Given the requirement for HA to rapidly undergo major conformational rearrangements at the precise moment a specific local environment is reached, the pre‐fusion form is capable of transiently exploring alternative conformations via molecular breathing [25, 26, 28, 29]. Its pre‐fusion form is described as being “meta‐stable”. The post‐fusion six‐helix bundle is its most energetically favorable conformation [25]. Because this conformation is irreversible, transient molecular events do not proceed fully to the post‐fusion conformation at appreciable rates. On virions and cell surfaces, HA samples conformations that expose interface epitopes [16, 17, 18, 19, 20, 21, 22, 23, 24].

The antibodies described below can stain the surfaces of cells expressing HA, even when it is in a precursor form that has not been processed by cellular proteases and therefore cannot adopt a post‐fusion conformation. HA trimers can tolerate rearrangements that expose interfaces within the head and stem while retaining a near‐prefusion form [30, 31].

## 3 The Head Interface/Trimer Interface

**FIGURE 2:**
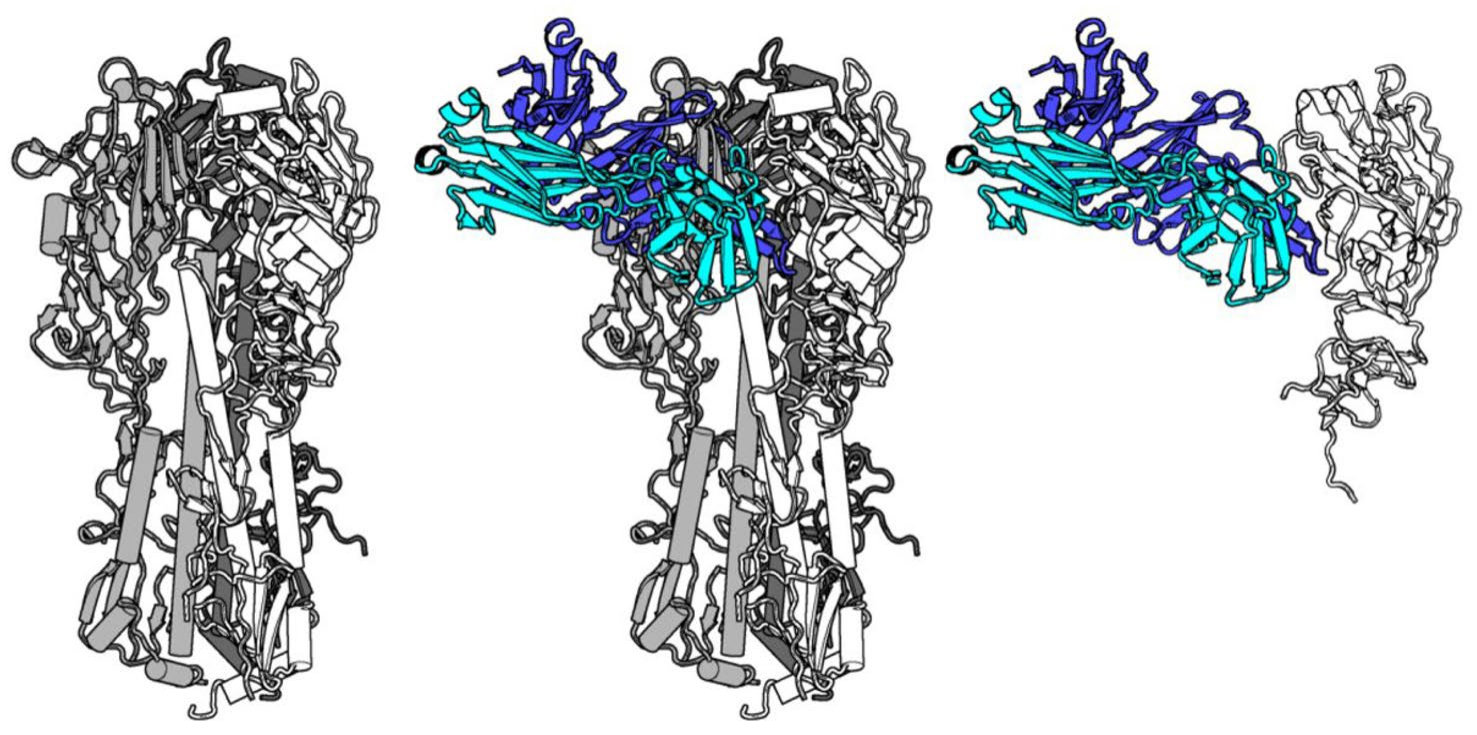
The head interface. Left: A pre‐fusion HA trimer (PDB 2VIU [6]); its protomers are colored from white to dark gray. Middle: Antibody S5V2–29 (PDB 6E4X [16]) modeled onto the trimer to highlight the requirement of a conformational rearrangement to expose its epitope. Its heavy chain is colored blue, and its light chain is colored cyan. Right: The structure of the S5V2–29 complexed with a monomeric HA head (PDB 6E4X).

Within the HA trimer, heads contact each other. These surfaces are widely conserved. Broadly binding and protective antibodies have been identified that engage a conserved surface at this HA head–head interface (Figure 2) [16, 17, 18, 23, 24]. The epitope for these antibodies is designated as the HA head interface or HA timer interface. The head interface is immunogenic in humans and in mice [16, 17, 18, 23, 24]. In humans, these antibodies often bind multiple, or even most, HA subtypes and seasonal antigenic variants. Head interface antibodies are present in circulating B cell repertoires (memory, plasma and bulk peripheral blood mononuclear cells (PBMCs)) following experimental infection, vaccination and at steady state [16, 17, 18, 23, 24, 32, 33]. In some adult humans, antibodies to the head interface can dominate the HA‐specific antibody responses in the serum repertoire [33]. Head interface antibodies are also elicited in murine models, and their abundance can be enhanced by selective immunogens [17].

The human antibody repertoire to the head interface harbors a genetically similar, public population and a genetically diverse population. Approximately half of the described antibodies to this site use an IGκV1‐39*01 or an identical duplicated copy IGκVD1‐39 [16, 18, 23, 24]. Affinity maturation selects for a characteristic S53N substitution. These light chains are most frequently paired with an IGHV4‐61*01 or similar heavy chain (e.g., IGHV4‐59*01). Restriction for the heavy chain is not absolute and is not restricted to IGHV4 family members. Antibodies from the IGκV1‐39*01 class have HCDR3s ≥ 18 amino acids in length with no reported constraints on light chain CDR3 lengths. All reported heavy chains have an acidic residue in the 6th position of HCDR3 [16, 18, 23, 24]. While these antibodies engage HA similarly, affinity maturation can produce B cell clones that are differentially impacted by antigenic variation [16, 18, 23, 24, 34].

Humans can also mount a genetically diverse antibody response to the head interface [16, 18]. In the B memory cell repertoire, these account for approximately half of the described examples. The angles of approach and how heavy and light chains contact the head interface can be quite distinct. While they approach the head interface differently and make differing series of contacts, they often share a common, overlapping footprint [16, 18]. The presence of these antibodies indicates that there are multiple antibody gene usages and affinity maturation pathways available to produce broadly binding, broadly protective antibodies. Antibodies with both relatively long or short CDR3s can engage the head interface. Perplexingly, all reported antibody examples utilize a kappa light chain. Structural analysis of more than a dozen antibodies has yet to identify an explanation for the strong bias for kappa light chains.

Mice also mount head interface‐directed responses [17]. Thus far, antibodies using IGHV5‐9‐1*02 paired with IGLV1*01 have been described. These antibodies have considerable breadth of binding for H3 isolates (the eliciting subtype). Modification of HA with additional glycans to focus antibody responses to conserved sites can boost the proportion of antibodies to the head interface and represent a viable strategy to seed antibody repertoires with interface‐directed antibodies, alongside those to additional conserved sites that are bound by broadly protective antibodies [17].

## 4 Head‐Stem Interface

**FIGURE 3:**
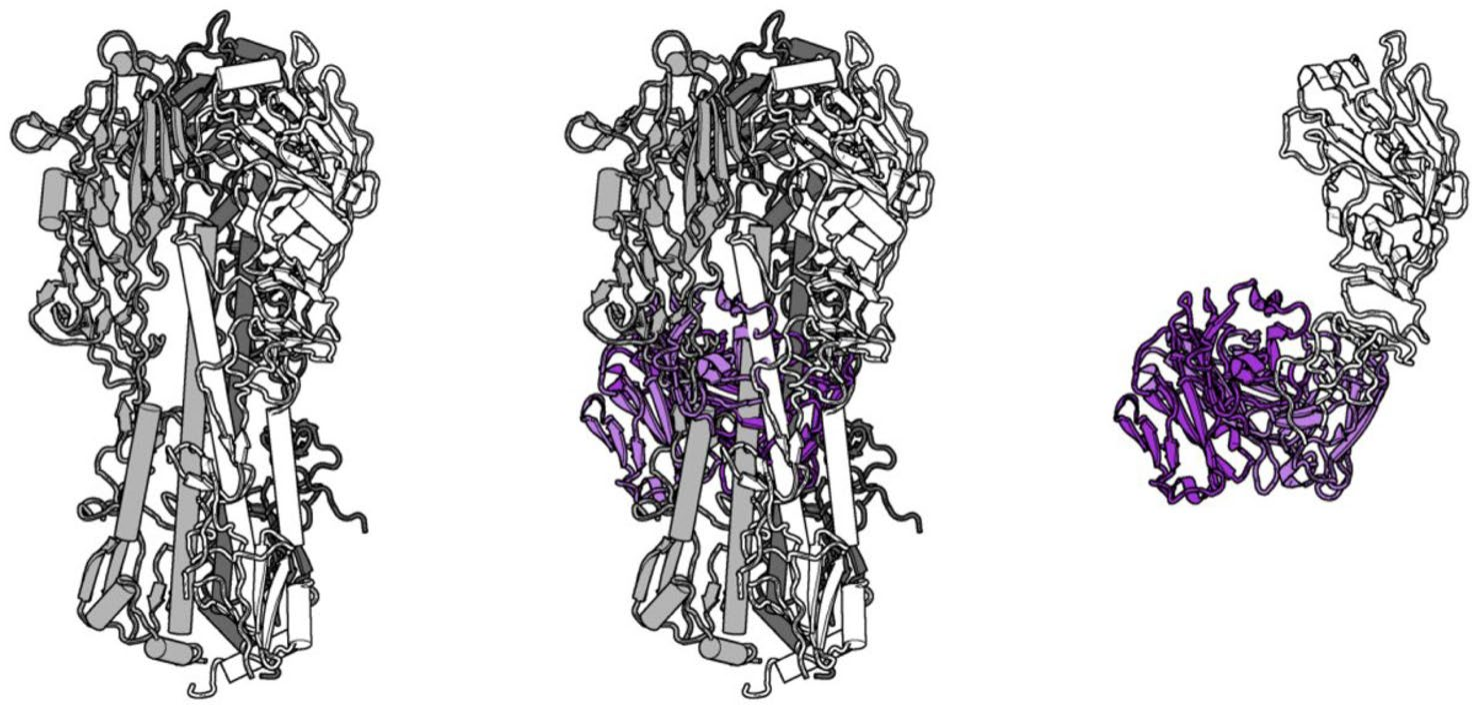
The head‐stem interface. Left: A pre‐fusion HA trimer (PDB 2VIU [6]); its protomers are colored from white to dark gray. Middle: Antibody S8V1–157 (PDB 8US0 [19]) modeled onto the trimer to highlight the need for the head and stem regions to separate to expose its epitope. Its heavy chain is colored purple, and its light chain is colored light purple. Right: The structure of the S8V1–157 complexed with a monomeric HA head (PDB 8US0).

Antibodies have recently been described that engage a conserved surface on the HA head at its interface with the HA stem (Figure 3) [19]. Antibody examples to this head‐stem interface have been identified in multiple human donors and account for at least ~1% of the influenza‐specific circulating B memory repertoire [16, 19]. Reported head‐stem interface antibodies engage representatives of most HA subtypes and human seasonal influenza variants. When passively transferred, these antibodies confer protection against fatal outcomes and ameliorate disease. Protection is enhanced when delivered with an Fc region that can direct cellular and complement‐mediated cell killing [19].

The canonical antibodies of this class utilize an IGKV2‐28*01 light chain, or an identical IGKV2D‐28*01 [19]. These are most frequently paired with IGHV2‐24*01 or IGHV5‐51*01. These antibodies share short HCDR3s (11–13 amino acids) and LCDR3s of 9–10 amino acids in length. Antibodies to the head‐stem interface sharing these features have been isolated from at least three human donors and are likely present in most human antibody repertoires [19].

A screen of single B cell cultures identified additional antibodies that compete for HA binding with head‐stem interface antibodies [19]. In addition to IGKV2‐28*01 antibodies with the features outlined above, a subset of genetically diverse antibodies was identified from three individuals. No apparent restrictions were found on IGVH gene usage or HCDR3 lengths. Like the head interface, all light chains used kappa light chains, but these were from different gene families [19]. These observations indicate that humans are capable of mounting head‐stem interface antibody responses with limited genetic restriction.

The head‐stem interface is immunogenic and bound by antibodies belonging to a genetically restricted, public clonotype and by genetically diverse antibodies [19]. Humans have the genetic capacity to produce both types of antibodies. Additional study is required to determine whether improved immunogens should be designed to elicit strong responses to the head‐stem interface.

## 5 Beta‐Hairpin

**FIGURE 4:**
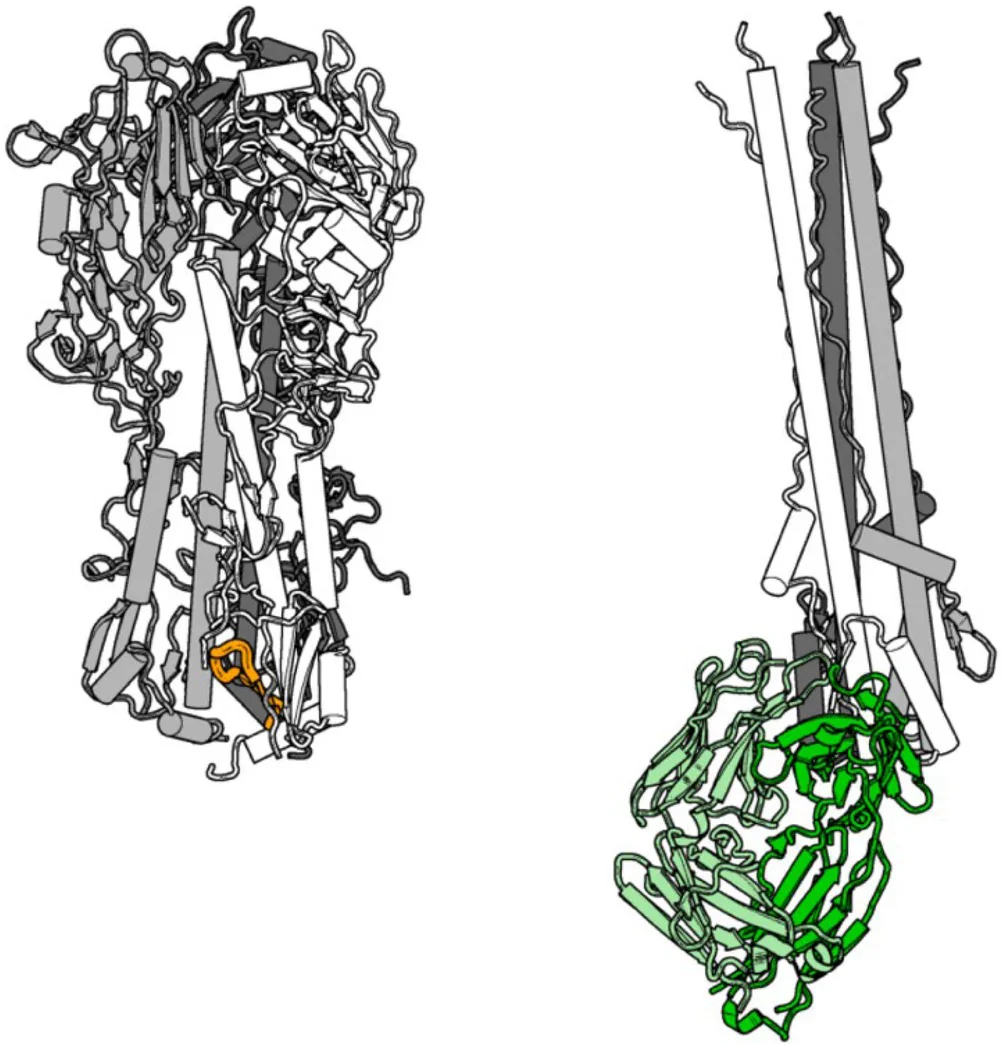
The beta‐hairpin. Left: A pre‐fusion HA trimer (PDB 2VIU [6]); its protomers are colored from white to dark gray. The beta‐hairpin that is bound by these antibodies is highlighted in orange. Right: The structure of the S1V2–72 complexed with a post‐fusion HA (PDB 8UDG [20]). Its heavy chain is colored green, and its light chain is colored light green. Transition of HA from pre‐fusion to post‐fusion states converts this occluded epitope into one that is solvent‐exposed.

A recently reported class of antibodies has been found to engage a beta hairpin in the HA stem (Figure 4) [20]. These antibodies, described as beta‐hairpin antibodies, can bind multiple influenza A subtypes, antigenic variants within these subtypes, and influenza B HAs. Antibodies to this site have reportedly used IGHV1‐2*02 paired with IGLV2‐23*02. Antibodies to the stem beta‐hairpin with this genetic signature have been identified in at least six human donors [20].

Unlike most of the stem, the beta‐hairpin is present in both the pre‐fusion and post‐fusion conformation [8, 20, 27]. The one reported structure of a beta‐hairpin antibody complexed with HA is its post‐fusion form [20]. In biolayer interferometry experiments, affinity is highest for post‐fusion HAs. HA binding experiments were also performed using HAs that were not processed by cellular proteases and cannot transition to a post‐fusion conformation [20, 25]. In Luminex or cell‐surface staining experiments, beta‐hairpin antibodies engaged these HAs. It may therefore be possible that these antibodies can engage HAs in multiple conformational states.

Passively transferred stem beta‐hairpin antibodies protect research animals from severe disease. Protection by these antibodies is greatly enhanced when their Fc‐region can direct cellular and complement‐mediated cell killing. Antibodies to this site, whether to the HA pre‐fusion or post‐fusion form, are likely present in most influenza‐exposed human repertoires. Should stem beta‐hairpin antibodies prove beneficial to improved immunogen designs, their immunogenicity is likely.

## 6 Long Alpha Helix

**FIGURE 5:**
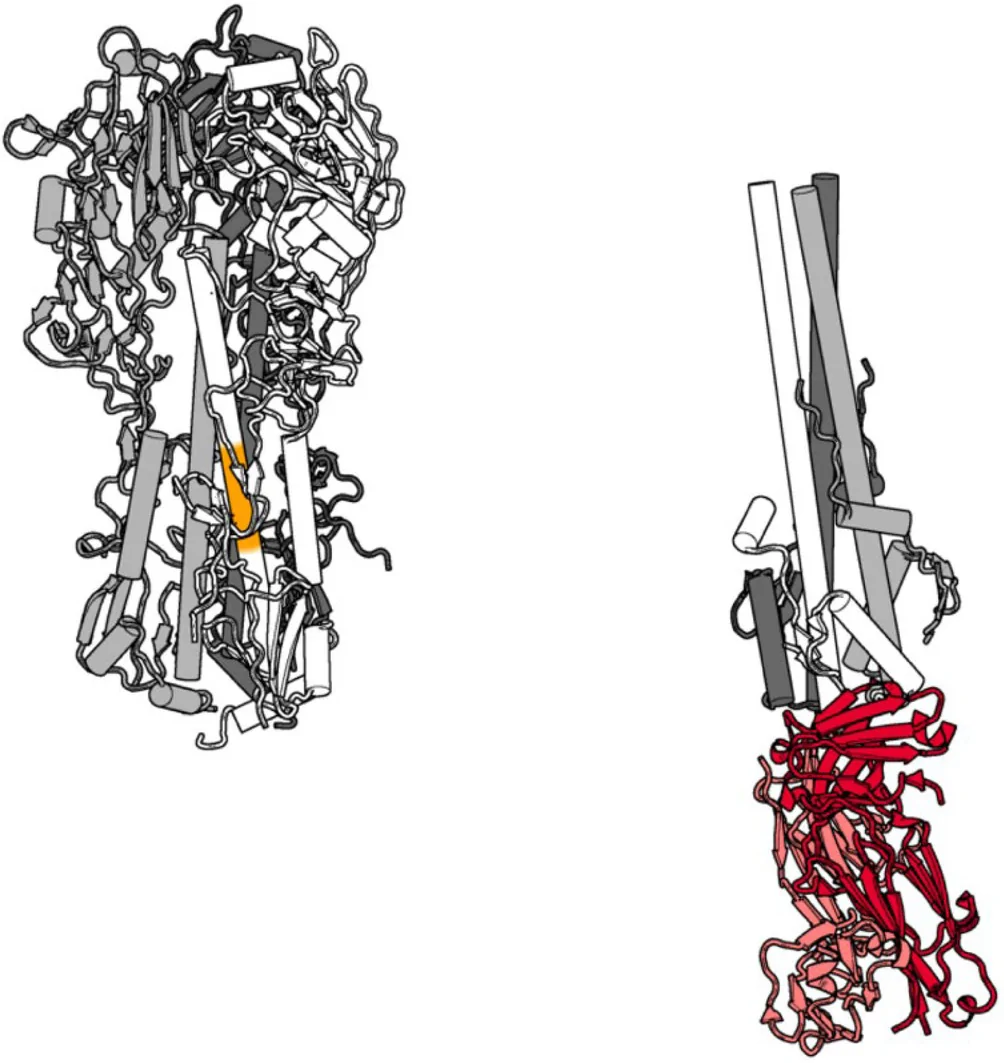
The long alpha helix. Left: A pre‐fusion HA trimer (PDB 2VIU [6]); its protomers are colored from white to dark gray. The long alpha helix is highlighted in orange and is buried within the HA trimer. Right: The structure of LAH31 complexed with a post‐fusion HA (PDB 8IB1 [22]). Its heavy chain is colored red, and its light chain is colored pink. The transition of HA from pre‐fusion to post‐fusion states converts an occluded epitope into a solvent‐exposed epitope.

Mice that have recovered from experimental influenza infection and adult humans that have likely been infected and given influenza vaccines harbor antibodies to the long alpha helix (LAH) within the HA stem (Figure 5) [21]. In mice, antibodies to the LAH can be dominant. Thus far, no genetic signature for LAH antibodies has been identified [21, 22]. Human and murine LAH antibodies have the highest affinity for HA in its post‐fusion form. These antibodies also bind peptides that correspond to regions of the long alpha helix. At least some of these antibodies can engage their LAH epitope on HA even under denaturing conditions in western blots [35]. In the one reported structure of a human LAH antibody complexed with a peptide, the peptide adopts a post‐fusion conformation.

Like other interface antibodies, these antibodies are broadly reactive across HA subtypes and antigenic variants within subtypes [21, 22]. Protection by these antibodies is enhanced by the ability to direct Fc‐mediated cell‐killing activities. Immunization of mice with post‐fusion HA can also elicit antibodies to the LAH that are protective in passive transfer studies.

LAH antibodies make the strongest case that post‐fusion forms of HA are efficiently presented to B cells. Whether virions or cell‐expressed HA have a subset of trimers that have transitioned to a post‐fusion form, infected cells display/present HA trimers after they have trafficked through low‐pH compartments, or HAs presented to B cells in germinal centers accumulate in this state is currently unknown. Understanding how HA is presented to B cells and the forms it adopts is critical to the design of improved immunogens.

## 7 Implications

HA interface epitopes are immunogenic in humans. At least a subset of sites are immunogenic and are present in murine models. Humans have the genetic capacity to mount responses to a spectrum of interface/post‐fusion surfaces. Considering the abundance and diversity of interface‐directed antibodies, obtained “accidentally” (as the HA probes used to isolate and assess binding to them were believed to be in the prefusion form) from a small number of human donors, it is likely that most influenza‐exposed humans mount antibody responses to interface epitopes. In some donors, interface antibodies are the dominant response to HA. In this review, we focused on interfaces that are bound by a number of broadly binding antibodies. We acknowledge that we have not comprehensively described every antibody to every interface epitope. Given the number of reported antibodies, particularly those on non‐human HAs (e.g., m826 [36] and H7.200 [37]), the size of the interface antibody repertoire is likely greater than currently understood. Establishing whether interface antibodies represent an antigenic “bug” or “feature” is a critical, unresolved question for designing improved influenza immunogens.

The abundance of interface antibodies contrasts with some of the assumptions of rational immunogen design. Candidate, improved immunogens for influenza viruses are designed to present the static pre‐fusion form of HA. In this form, interface epitopes are occluded. The presence of interface antibodies indicates that there is a disconnect between our structural understanding of viral/vaccine antigens or designed immunogens that are produced during infection or delivered in vaccinations and ultimately what is presented to B cells. Resolving this knowledge gap is critical.

## Conflicts of Interest

The author declares no conflicts of interest.

## Data Availability

Data sharing not applicable to this article as no datasets were generated or analyzed during the current study.
